# Supplementary material for: Comparison between Immunocytochemistry, FISH and NGS for ALK and ROS1 Rearrangement Detection in Cytological Samples
Source: Int J Mol Sci. 2022 Sep 12;23(18):10556. doi: 10.3390/ijms231810556 (PMC9502752; doi:10.3390/ijms231810556)
Supplement: Supplementary file 1 [file ijms-23-10556-s001.zip › Table S1.pdf]

Table S3. Samples repartition.

	ALK			ROS1		
**Cytological sample specimen types**	**NGS**	**ICC**	**FISH***	**NGS**	**ICC**	**FISH***
**EBUS-TBNA lymph nodes (n=59)**	Neg = 47<br>Pos = 6<br>NE = 6	Neg = 35<br>Pos (+ to +++) = 5<br>NI = 2<br>NP = 17	Neg = 2<br>Pos n = 4<br>NE = 2	Neg = 53<br>NE = 6	Neg = 41<br>NI = 2<br>NP = 16	Neg = 2
**Pleural (n=45)**	Neg = 35<br>Pos = 9<br>NE = 1	Neg = 27<br>Pos (+ to +++) = 12<br>NI = 2<br>NP = 4	Neg = 8<br>Pos = 9	Neg = 41<br>Pos = 3<br>NE = 1	Neg = 30<br>Pos (+ to +++) = 10<br>NI = 1<br>NP = 4	Neg = 8<br>Pos = 2
**EBUS-TBNA mediastinal or pulmonary mass (n=9)**	Neg = 9	Neg = 4<br>Pos (+ to +++) = 1<br>NP = 4	Neg = 1	Neg = 9	Neg = 4<br>Pos (+ to +++) = 1<br>NP = 4	Neg = 1
**Pericardial (n=7)**	Neg = 7	Neg = 7		Neg = 7	Neg = 4<br>Pos (+ to +++) = 3	Neg = 3
**Bronchial brush (n=5)**	Neg = 5	Neg = 4<br>NP = 1	/	Neg = 5	Neg = 3<br>Pos (+ to +++) = 1<br>NP = 1	Neg = 1
**BAL (n=4)**	Neg = 4	Neg = 2<br>NP = 2	/	Neg = 4	Neg = 2<br>NP = 2	/
**CSF (n=2)**	Neg = 1<br>NE = 1	NP = 2	/	Neg = 1<br>NE = 1	NP = 2	/
**Total (n=131)**	**Neg = 108**<br>**Pos = 15**<br>**NE = 8**	**Neg = 79**<br>**Pos (+ to +++) = 18**<br>**NI = 4**<br>**NP = 30**	**Neg = 11**<br>**Pos = 13**<br>**NE = 2**	**Neg = 120**<br>**Pos = 3**<br>**NE = 8**	**Neg = 84**<br>**Pos (+ to +++) = 15**<br>**NI = 3**<br>**NP = 29**	**Neg = 13**<br>**Pos = 2**

ICC: Immunocytochemistry; FISH: Fluorescence *in situ* hybridization; NGS: Next Generation Sequencing; NE: Not exploitable; Neg: negative; Pos: positive; NP: not performed; NI: not interpretable. *FISH was only performed when the result of ICC was + to +++ and when the material was still available.
