# Supplementary figures and images for: Comparison between Immunocytochemistry, FISH and NGS for ALK and ROS1 Rearrangement Detection in Cytological Samples
Source: Int J Mol Sci. 2022 Sep 12;23(18):10556. doi: 10.3390/ijms231810556 (PMC9502752; doi:10.3390/ijms231810556)

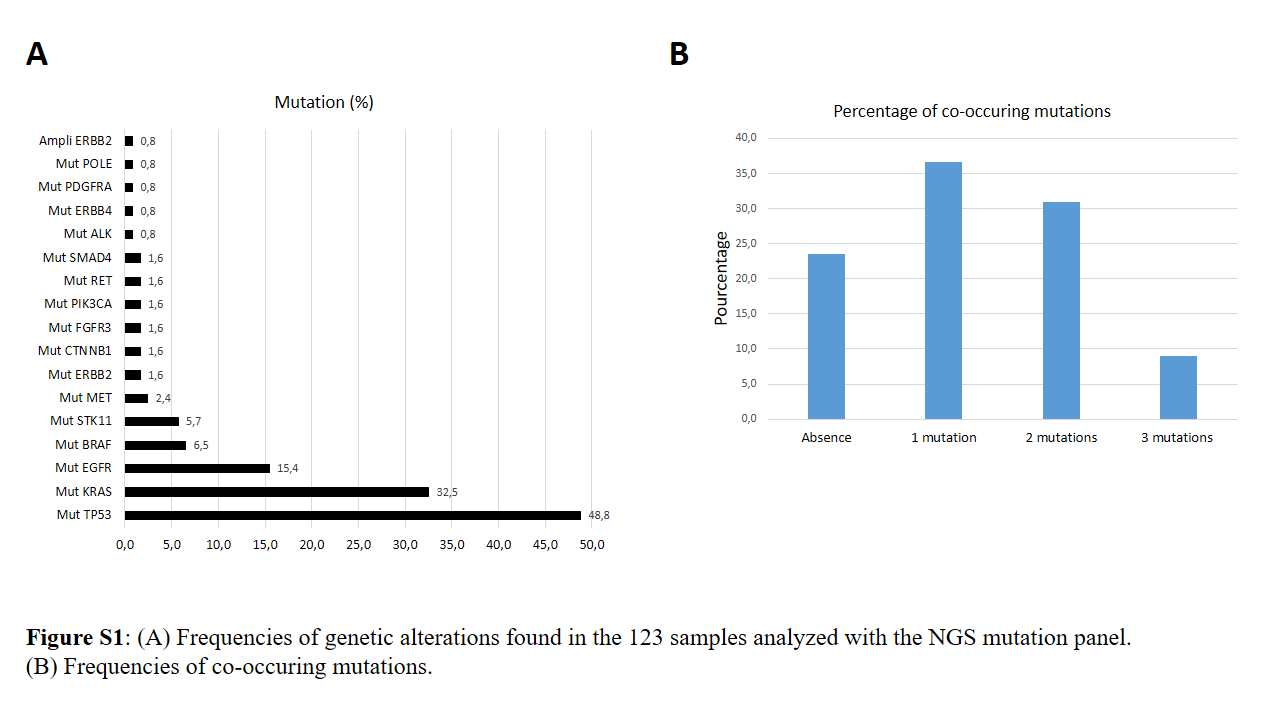

Supplement: Supplementary file 1 [file ijms-23-10556-s001.zip › Figure S1.tif]
